# Talin-1 variants associated with spontaneous coronary artery dissection (SCAD) highlight how even subtle changes in multi-functional scaffold proteins can manifest in disease

**DOI:** 10.1093/hmg/ddae120

**Published:** 2024-08-21

**Authors:** Latifeh Azizi, Yasumi Otani, Vasyl V Mykuliak, Benjamin T Goult, Vesa P Hytönen, Paula Turkki

**Affiliations:** Faculty of Medicine and Health Technology, Tampere University, Arvo Ylpön katu, 33520 Tampere, Finland; Department of Biochemistry, Cell & Systems Biology, Institute of Systems, Molecular & Integrative Biology, University of Liverpool, Crown Street, Liverpool L69 7ZB, United States; Faculty of Medicine and Health Technology, Tampere University, Arvo Ylpön katu, 33520 Tampere, Finland; Department of Biochemistry, Cell & Systems Biology, Institute of Systems, Molecular & Integrative Biology, University of Liverpool, Crown Street, Liverpool L69 7ZB, United States; Faculty of Medicine and Health Technology, Tampere University, Arvo Ylpön katu, 33520 Tampere, Finland; Fimlab Laboratories, Biokatu 4, 33520 Tampere, Finland; Faculty of Medicine and Health Technology, Tampere University, Arvo Ylpön katu, 33520 Tampere, Finland; Fimlab Laboratories, Biokatu 4, 33520 Tampere, Finland

**Keywords:** talin-1, SCAD, I25.42, variant classification, TLN1, pathogenicity prediction

## Abstract

Variants of talin-1 (*TLN1*) have recently been linked with spontaneous coronary artery dissection (SCAD) a condition where a tear can form in the wall of a heart artery necessitating immediate medical care. One talin-1 variant, A2013T, has an extensive familial pedigree of SCAD, which led to the screening for, and identification of, further talin-1 variants in SCAD patients. Here we evaluated these variants with commonly used pathogenicity prediction tools and found it challenging to reliably classify SCAD-associated variants, even A2013T where the evidence of a causal role is strong. Using biochemical and cell biological methods, we show that SCAD-associated variants in talin-1, which would typically be classified as non-pathogenic, still cause a measurable impact on protein structure and cell behaviour, including cell movement and wound healing capacity. Together, this indicates that even subtle variants in central mechanosensitive adapter proteins, can give rise to significant health impacts at the individual level, suggesting the need for a possible re-evaluation of the scoring criteria for pathogenicity prediction for talin variants.

## Introduction

**Figure 1:**
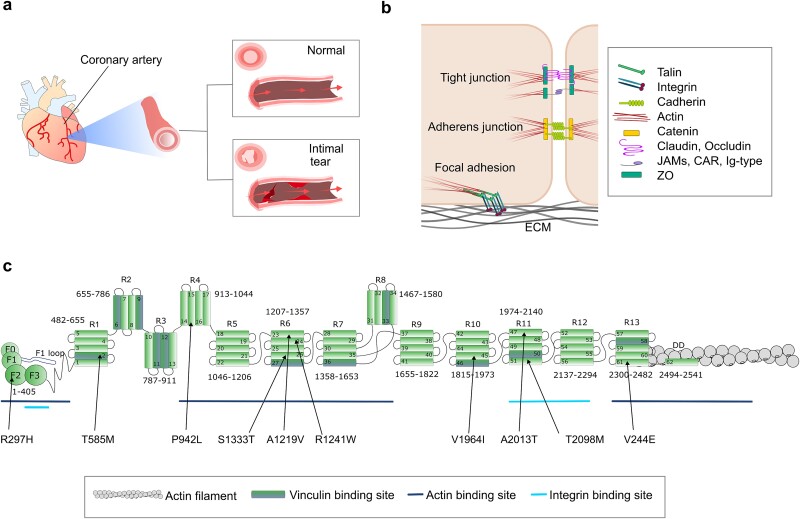
SCAD-associated talin-1 variants. (a) Schematic representation of SCAD, where a sudden rupture of the coronary arterial wall occurs, resulting in an intimal tear. (b) Arteries are comprised of an endothelial cell monolayer. Both cell–cell and cell-ECM junctions are required to maintain the endothelial cell monolayer integrity. (c) Schematic domain structure of talin-1, comprising an N-terminal head domain (subdomains F0-F3), rod domain (R1-R13), and dimerisation domain (DD) with the location of each talin-1 SCAD-associated variant labelled. Each rod domain is a bundle of 4 or 5 helices. The binding sites for vinculin (indicated as darker helices in the domain, with a total of 11 binding sited located across various regions), integrin (underlinedin subdomains F3 and R11-R12) and actin (underlined in the head domain, R4-R8 and R13-DD) are highlighted. An F-actin filament is shown connecting to the C-terminal actin binding domain.

The intima, the innermost layer of an artery or vein, consists of a monolayer of endothelial cells connected to a collagen-rich elastic layer. These endothelial cells are essential in maintaining the integrity of the intima, as they serve as the primary defence barrier helping to protect the vessel from mechanical stresses (Fig. 1a). Endothelial cells possess two major types of cell adhesions: cell–cell junctions between the neighbouring cells, and cell-extracellular matrix (ECM) adhesions to the underlying protein meshwork (Fig. 1b). Adjacent endothelial cells are connected to each other by two types of intercellular junctions, tight junctions and adherens junctions. Tight junctions are located near to the apical side of the cells and are comprised of transmembrane proteins such as claudin, occluding and junction adhesion molecule (JAM) and intracellular adaptor proteins such as Zonula Occludens-1 (ZO-1) connecting these to the cytoskeleton [1]. Adherens junctions are mediated by cadherin receptors, which form homophilic interactions between the extracellular domains of cadherins on neighbouring cells. The short intracellular regions of the cadherins scaffold intracellular protein complexes containing catenins, that connect to the actin cytoskeleton [1]. In the basal side, cell-ECM adhesions called focal adhesions are organized around integrin receptors, which connect to the ECM on the extracellular side and the short cytoplasmic tails of the integrins scaffold intracellular protein complexes, that connect to the actin cytoskeleton [2]. All of these adhesion complexes respond to forces and influence the cytoskeleton, particularly the actin network [3, 4]. Whilst the junctions between endothelial cells are impermeable, leukocytes are able to migrate between them to transmigrate from the vessel into the tissue. This transendothelial migration, a hallmark of the tissue inflammation process, is mediated via another mode of cell–cell junction formation, where leukocytes adhere to the endothelial cells.

The dynamic interplay between cell–cell and cell-ECM adhesions plays a crucial role in regulating blood vessel development and stability during angiogenesis, inflammation and shear stress [5]. Integrin acetylation was recently shown to lead to immature cell-ECM adhesions modulating endothelial cell–cell junction integrity, contact inhibition and transcription level changes of genes associated with endothelial barrier function [6]. In addition to endothelial cell function, abnormalities in integrin and cadherin interactions can result in impaired insulin secretion and abnormal islet morphology, contributing to diabetes [7–10]. Furthermore, dysregulation of apoptosis and epithelial-mesenchymal transition can contribute to the progression and metastasis of cancer [11–14]. These examples demonstrate how interruptions in the balance of these adhesion systems can lead to various diseases.

Talins (talin-1 and talin-2) play a central role in the formation of integrin-mediated cell-ECM adhesions. As a key integrin regulator, talin contributes to integrin activation, influencing their binding to the ECM. Talin binds to the short cytoplasmic tail of the integrin beta-subunit and connects it to the actin cytoskeleton by interacting directly, and indirectly, with actin. By modulating integrin activation, talin influences the intricate balance between cell-ECM and cell–cell adhesion, contributing to the maintenance of endothelial barrier function. In endothelial cells, talin is also involved in the stability and organization of Vascular Endothelial cadherin (VE-cadherin), a key component of adherens junctions [15], identifying talin as a molecular coordinator, bridging the communication gap between integrins and cadherins to regulate cellular interactions and ensure endothelial integrity. Evidence of this bridging role between cell–cell and cell-ECM adhesions is evident from the identification of a mutation in the talin-1 (*TLN1*) gene that gives rise to systemic capillary leak syndrome (SCLS). Here a single nucleotide mutation in *TLN1* leads to in-frame skipping of exon 54 affecting the C-terminal actin-binding domain in talin, which results in the cadherin-mediated adherens junctions becoming disorganised [16].

Spontaneous Coronary Artery Dissection (SCAD) is a relatively infrequent, yet potentially life-threatening condition that can lead to ischemia and acute myocardial infarction. While SCAD accounts for approximately 0.2%–4% of all acute coronary syndrome (ACS) cases, it is not uniformly distributed across all patient groups. Notably, SCAD is most prevalent among women, especially those around the age of 50. In this specific subgroup, the prevalence of SCAD can escalate to between 22.5%–35% of ACS cases, highlighting the importance of considering demographic factors in the diagnosis and management of ACS [17, 18]. Studies have identified some of the predisposing factors for SCAD including fibromuscular dysplasia, hormonal therapy, multiparity, connective tissue disorders, and systemic inflammatory diseases [17, 19, 20]. Furthermore, emerging evidence shows that cardiogenic shock, ventricular arrhythmia, and cardiac arrest are common, with a higher incidence of complications among peripartum women [17, 21]. However, there is an incomplete understanding of the genetic risk factors contributing to SCAD.

To identify genetic factors behind SCAD, Turley *et al*. conducted whole-exome sequencing on a family where four individuals across two generations of the family were affected. This analysis identified a heterozygous missense variant, A2013T, within the *TLN1* gene [22]. While there are two main talin isoforms in humans, talin-1 is the exclusive form found in endothelial cells [23] and they conclusively linked the A2013T variant in talin-1 to SCAD [22]. Based on this confirmed disease association between a variant in *TLN1* and SCAD, they went on to screen 675 unrelated individuals diagnosed with SCAD and examine the frequency of *TLN1* variants in the cohort. This screening approach identified a further nine rare heterozygous missense talin-1 variants (R297H, T585M, P942L, A1219V, R1241W, S1333T, V1964I, T2098M, and V2440E) in the SCAD cohort. Notably, the A1219V variant was found in two unrelated individuals. Therefore, in total, ten unique talin-1 variants have now been identified that are associated with SCAD, including A2013T from the familial case (Fig. 1c).

In our previous studies, we explored the cellular consequences of cancer-associated somatic talin-1 mutations [24] and a unique talin-1 variant, P229S, which was found to be associated with diverse symptoms, including thrombocytopenia, lymphopenia, congenital cataracts, and skin changes [25]. These studies demonstrated that the impact of disease-associated mutations in talin-1 can be clearly measured both in terms of protein function at the biochemical level but also as measurable phenotypes at the cellular level leading to mild functional defects, such as impaired cell migration *in vitro*.

To gain insights into the role of talin-1 in SCAD pathogenesis, we conducted studies on the ten *TLN1* missense variants identified by Turley *et al*. Using a multidisciplinary approach, we integrated pathogenicity prediction, molecular dynamics simulations, biophysical methods and functional cell biology to determine the functional characteristics of these variants. Our findings reveal that SCAD-associated talin-1 variants, classified mostly as non-pathogenic by the widely used pathogenic prediction tools, still exert a measurable impact on protein properties and cellular functions in both fibroblasts and endothelial cells. Together, these observations indicate that even subtle variants in the central integrin adapter protein talin-1 can result in significant health impacts and warrants careful assessment of findings from clinical genomics.

## Results

### Prediction of functional consequences of SCAD-linked talin-1 variants

There are numerous algorithms available for predicting the influence of missense mutations. In this study, we incorporated six prediction tools for assessing pathogenicity (Table 1) to predict the effect of the SCAD-linked talin-1 variants. PolyPhen-2 [26, 27], Sorting Tolerant From Intolerant (SIFT) [28, 29], and Protein Variation Effect Analyzer (PROVEAN) [30] are conservation-based prediction tools, evaluating the consequence of mutations by considering similar proteins and local sequence conservation. We also used the Rare Exome Variant Ensemble Learner (REVEL) [31] and Individual Meta Random Forest (InMeRF) [32] algorithms, which, by combining meta-prediction and machine learning, have been shown to improve accuracy compared to methods based on sequence conservation alone. Finally, we included the most recent development in the field of structure-based prediction and evaluated how AlphaMissense [33] classifies the variants. AlphaMissense combines structural context and evolutionary conservation of the mutated position to predict the clinical consequences of missense variants [34].

**Table 1:** Pathogenicity prediction of SCAD-associated talin-1 variants using six different prediction tools. SIFT, PolyPhen-2, PROVEAN, REVEL, InMeRF and AlphaMissense were used to assess the potential impact of the SCAD-associated talin-1 variants. The P229S talin-1 variant [25] was also included as a reference.

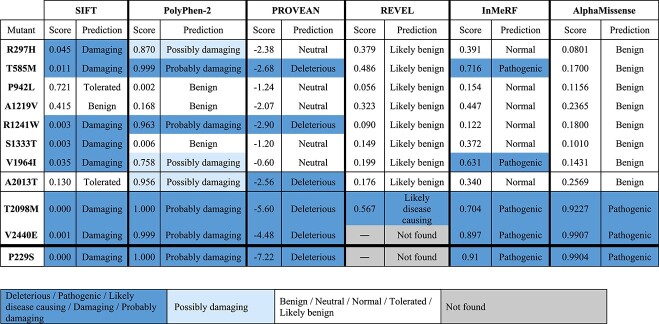

As summarised in Table 1, the SCAD-associated mutations yield rather divergent predictions depending on the bioinformatic pathogenicity prediction software used. While some variants (T585M, R1241W, T2098M, V2440E) are predicted as pathogenic by several tools, many are entirely overlooked by predictors. Surprisingly, the A2013T variant, traced through pedigrees to familial SCAD, is predicted as benign by several predictors including AlphaMissense. Only PolyPhen-2 and PROVEAN predicted this particular variant as deleterious, with relatively low scores.

The A1219V variant, identified in two unrelated individuals in the SCAD cohort [22], was consistently predicted as benign or neutral by all six prediction tools. Among the 10 variants, only T2098M and V2440E yielded consistent prediction to be deleterious (REVEL prediction was not available for V2440E).

From our previous study, the P229S variant associated with multifaceted clinical symptoms [25] is predicted as pathogenic by all the bioinformatic pathogenicity prediction tools (not available for REVEL) and was included here as a reference (Table 1).

### Structural assessment of SCAD-associated talin-1 variants

The pedigree-confirmed SCAD-causing talin-1 variant, A2013T, resides in the talin-1 rod domain R11 (Fig. 1c). T2098M is also in R11, but the other eight SCAD-associated variants exhibit a broad distribution across the talin molecule (Table 2; Fig. 1c). Structural inspection revealed that seven out of the ten SCAD-associated variants are on residues located on the surface of the protein domains, with three variants, A1219V, V1964I and T2098M, corresponding to buried residues within the talin domains (Table 2).

**Table 2:** Influence of SCAD-associated talin-1 variants on the domain stability predicted by molecular dynamics simulations and measured by biophysical methods. For each mutation the subdomain and position within the subdomains is indicated. The contribution of each mutation to the free energy of protein folding (ΔΔG) was calculated, positive values mean the mutant is stabilising, and negative energy values mean the mutant has a destabilising effect. NMR HSQC spectra were used to evaluate if the domain (WT and mutant) was folded. Circular dichroism (CD) was used to determine the melting temperature (T_m_) for the wild-type and mutated subdomain and the change in melting temperature is reported (ΔT_m_ = T_m_(mutant)—T_m_(WT)).

**Mutation**	**Sub-domain**	**Position**	**ΔΔG (kJ/mol) ± STD**	**NMR**	**CD** **WT T**_**m**_ **(°C)**	**CD mutant T**_**m**_ **(°C)**	**∆T**_**m**_ **(°C)**
**R297H**	F2	Surface	2.91 ± 0.16	–	–	–	–
**T585M**	R1	Surface	−4.27 ± 0.90	–	–	–	–
**P942L**	R4	Surface	−1.10 ± 3.54	Folded	46.2	53.2	7.0
**A1219V**	R6	Buried	−0.44 ± 0.81	–	75.3	–	–
**R1241W**	R6	Surface	6.67 ± 1.98	Folded	75.3	71.8	−3.5
**S1333T**	R6	Surface	3.57 ± 0.54	–	75.3	–	–
**V1964I**	R10	Buried	0.05 ± 1.23	–	–	–	–
**A2013T**	R11	Surface	0.43 ± 0.90	Folded	59.8	55.7	−4.1
**T2098M**	R11	Buried	−5.14 ± 3.64	Folded	59.8	55.3	−4.5
**V2440E**	R13	Surface	−11.22 ± 0.66	Folded	>80	>90	N/A

To assess whether the SCAD-associated variants impact the stability and folding of talin domains, we first conducted molecular dynamics (MD) simulations to calculate the effect of each variant on the thermodynamic stability of the corresponding talin-1 subdomain (Table 2). For each mutation we calculated the free energy changes of the wild-type domain compared to the mutant version using alchemical free energy calculations [35] where a positive energy value indicates a destabilising effect, and a negative value a stabilising effect. This computational analysis indicated that mutations R297H (F2; 2.91 ± 0.16 kJ/mol), and S1333T (R6; 3.57 ± 0.54 kJ/mol) have a mild destabilising effect on the talin rod domain they are contained within, and R1241W (R6; 6.67 ± 1.98 kJ/mol) a more significant destabilising effect. In contrast, a moderate stabilising effect was observed for T585M (R1; −4.27 ± 0.9 kJ/mol) and T2098M (R11; −5.14 ± 3.64 kJ/mol), while the strongest stabilising effect was seen for V2440E (R13; −11.22 ± 0.66 kJ/mol). P942L (R4; −1.1 ± 3.54 kJ/mol), A1219V (R6; −0.44 ± 0.81 kJ/mol), V1964I (R10; 0.05 ± 1.23 kJ/mol) and A2013T (R11; 0.43 ± 0.9 kJ/mol) do not significantly alter the stability of the subdomains. Strikingly, T2098M and V2440E not only show elevated pathogenicity scores (Table 1) but also exhibit substantial changes in structural stability (Table 2).

**Figure 2:**
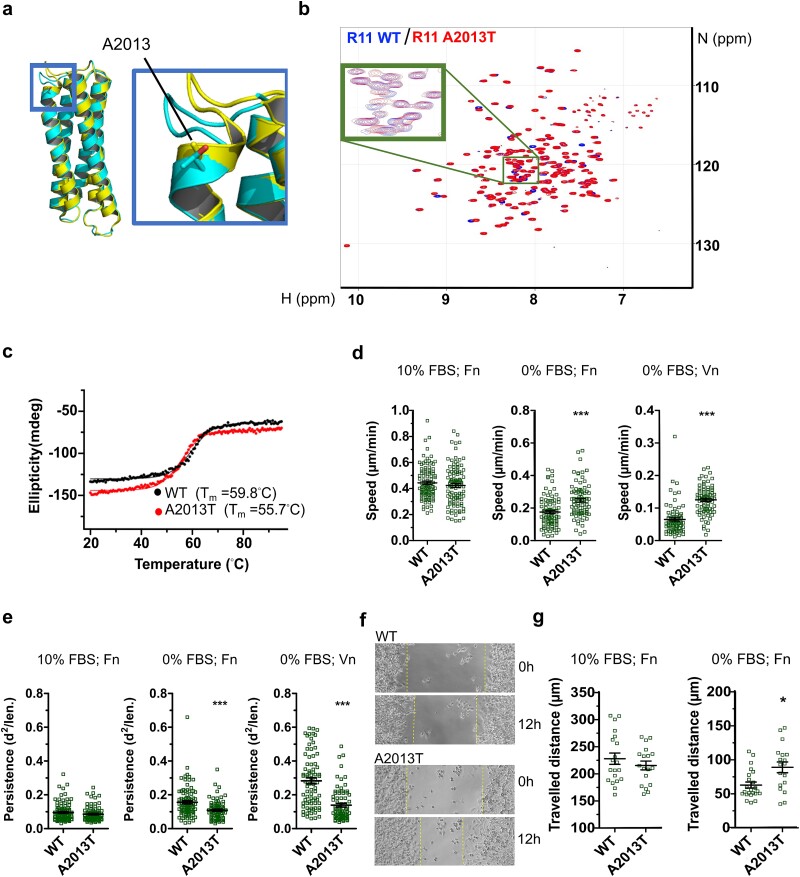
Biochemical and functional cell biology assessment of the A2013T variant in R11. This figure shows the full analysis of the A2013T variant. The analysis for the other SCAD-associated mutants can be found in Fig. S1. (a) Structure of talin-1 R11 WT (yellow, PDB 3DYJ [36], residues 1974–2140) and AlphaFold predicted structure model of talin-1 R11 A2013T (cyan). (b) 1H, 15N-HSQC spectra of 15N-labelled talin-1 R11 WT (blue) and A2013T (red). (c) Melting curves of talin-1 R11 WT (black) and A2013T (red) (d and e) TLN1 −/−TLN2 −/− MKF cells were transiently transfected with GFP-talin-1, either WT or A2013T, and the influence on the (d) random migration velocity and (e) persistence of cell movement was measured under different conditions; 10% FBS (fetal bovine serum) on fibronectin-coated substrate (Fn) (left), 0% FBS on Fn (middle) or 0% FBS on vitronectin (Vn). n ~ 80 cells pooled from three independent experiments. The statistical significance was analysed by one-way ANOVA and Bonferroni test: ^*^P < 0.05, ^*^^*^P < 0.01, ^*^^*^^*^P < 0.001. (f) Representative images of the wound closure assay for the cells transfected with full-length talin WT and A2013T at 0 h (upper) and after 12 h (lower) in 0% FBS medium (g) distance travelled (μm) by cells to close the artificial wound were calculated by analysing the travelled distance from 0–12 h for full-length talin WT and A2013T. n ~ 25 scratches from three independent experiments. The statistical analysis was done using t-test Mann–Whitney test; ^*^P < 0.05, ^*^^*^P < 0.01, ^*^^*^^*^P < 0.001. Data represent the mean values with SEM.

To experimentally evaluate the influence of the variants on domain folding and protein stability, we recombinantly expressed mutated talin rod domains and their wild-type counterparts (R4 +/− P942L, R6 +/− R1241W, R11 +/− A2013T, R11 +/− T2098M, and R13 +/− V2440E). In each case the mutated domains expressed well in *E. coli* with similar protein levels to their wild-type counterparts. Nuclear Magnetic Resonance (NMR) is a powerful tool for evaluating mutations in proteins, as the spectra provide a fingerprint of the domain, enabling evaluation of how well folded a domain is, and how it is affected by mutation. Comparison of the ^1^H,^15^N-HSQC (Heteronuclear Single Quantum Coherence) spectra of wild-type and mutated forms of each domain indicated that all domains folded efficiently with the spectral changes in the mutant spectra being modest as compared to WT spectra, confirming each mutated domain was still correctly folded (Fig. 2a and b; Fig. S1a–d). These findings are in line with the rather modest influence of the mutations thermodynamic stability analysed with MD simulations (Table 2).

Next, to investigate the influence of each mutation on talin domain thermal stability, we used circular dichroism (CD) with thermal scanning. Here the CD at 222 nm (maximal alpha-helix signal) is measured as the temperature is increased. As the domain unfolds, this signal is decreased, enabling the melting temperature (T_m_) to be determined. R4 with the P942L mutation showed significantly higher thermal stability than wild-type R4 (ΔT_m_ = 7.0°C, Table 2; Fig. S1e). P942 is at the very end of a helix, and mutation of P942L increases the thermal stability indicating that a proline capping the end of helix might have a role in the unfolding/refolding properties of the domain. Interestingly, many talin helices are capped by a proline residue in this fashion, suggesting that capping proline’s may have an important role in talin domain function. R1241W made R6 less stable (ΔT_m_ = −4.5°C), A2013T made R11 less stable (ΔT_m_ = −4.1°C) and T2098M was also destabilising in R11 (ΔT_m_ = −4.5°C) with a similar impact as A2013T (ΔT_m_ = −4.1°C) (Table 2; Fig. 2c; Fig. S1f and g). R13 rod domain is so thermally stable that it only starts unfolding at 80–90°C and is not completely unfolded even at 95°C, the upper temperature limit of the experiment. Therefore, the effect of the V2440E variant could not be measured accurately but V2440E was also highly stable with T_m_ > 90°C (Table 2; Fig. S1h).

### SCAD-associated talin-1 variants cause detectable changes in fibroblast migration

To investigate the effects of the SCAD-associated talin-1 variants on cell-ECM adhesion and cell motility, we transfected talin double knockout (*TLN1* −/− *TLN2* −/−) mouse kidney fibroblast (MKF) cells [37] with wild-type and variant versions of GFP-full-length talin-1. These cells rely on talin re-expression for adhesion and migration, making them an excellent tool to detect even subtle changes in talin function [24] without compensation from endogenous talin-1 and talin-2. To reveal the potential impact of the variants on cell migration speed, we conducted experiments in the presence or absence of Fetal Bovine Serum (FBS) and on either fibronectin (Fn) or vitronectin (Vn) coated coverslips. These different conditions can be used to mimic different conditions seen *in vivo* [38–40].

We first looked at the behaviour of the pedigree-confirmed SCAD-causing talin-1 variant, A2013T. In 10% FBS-containing medium, no changes in cell migration were observed in comparison to the wild-type. As cell adhesion is strongly influenced by the FBS components, we next performed the experiment in minimal serum concentration. In the absence of FBS and on both Fn and Vn, the A2013T talin-1 transfected cells showed enhanced cell migration (*P* < 0.001) compared to the wild-type (Fig. 2d).

We next repeated these experiments for the other nine SCAD-associated mutations. In the presence of 10% FBS with Fn coating, only the V2440E variant exhibited enhanced cell migration. In the absence of serum (0% FBS) the R1241W, A2013T, T2098M, and V2440E variants all resulted in increased cell migration on Fn. Furthermore, mutations P942L, S1333T, A2013T, and T2098M led to increased cell migration speed compared to wild-type talin when cells in 0% FBS were plated on substrate coated with Vn (Table 3; Fig. S2a–c).

**Table 3:** Impact of SCAD-associated talin-1 variants on random and collective cell migration. *TLN1* −/−*TLN2* −/− MKF cells were transiently transfected to assess how talin-1 mutations influence two aspects of cell migration. The random migration velocity (μm/min) of the cells. Collective migration (μm) for the distance travelled by cells to close an artificial wound. Data are mean ± s.d. The statistical significance of all results was analysed by one-way ANOVA and Bonferroni test. ^*^P < 0.05, ^*^^*^^*^P < 0.001. The full data for all the SCAD-associated mutants can be found in Fig. S2.

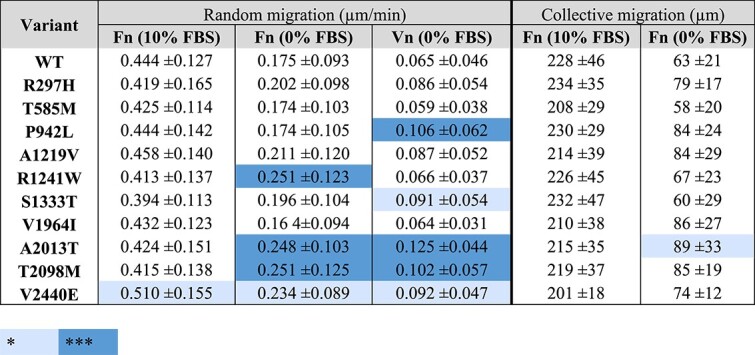

Cells cultured in FBS-reduced conditions can in principle form molecular gradients into the substrate which can guide the cell migration direction [41]. Without the masking effect of FBS, cells can then recognize the gradients and have persistence in their movement. We therefore measured if cells expressing A2013T mutant have differences in their persistency of movement when compared to WT expressing cells. Indeed, while in full-serum conditions poor directionality was observed, in serum-depleted conditions WT expressing cells were migrating more directionally compared to those expressing A2013T mutant (Fig. 2e). These results indicate that the cells expressing A2013T mutant either have defects in the matrix remodelling capacity or in the directional movement.

Next, we assessed the influence of these mutations on the ability of fibroblasts to respond to tissue defects using a wound healing assay *in vitro*. When cultivated in 10% serum, all mutants behaved similarly to the wild-type (Table 3; Fig. S2d and e). However, in serum-depleted conditions, A2013T showed significantly accelerated collective migration (Fig. 2f and g; Table 3). Additionally, we observed subtle, though not statistically significant, acceleration of collective cell migration with mutations R297H, P942L, A1219V, V1946I, T2098M, and V2440E (Fig. S2e).

### SCAD-associated talin-1 variants modify endothelial cell behaviour

**Figure 3:**
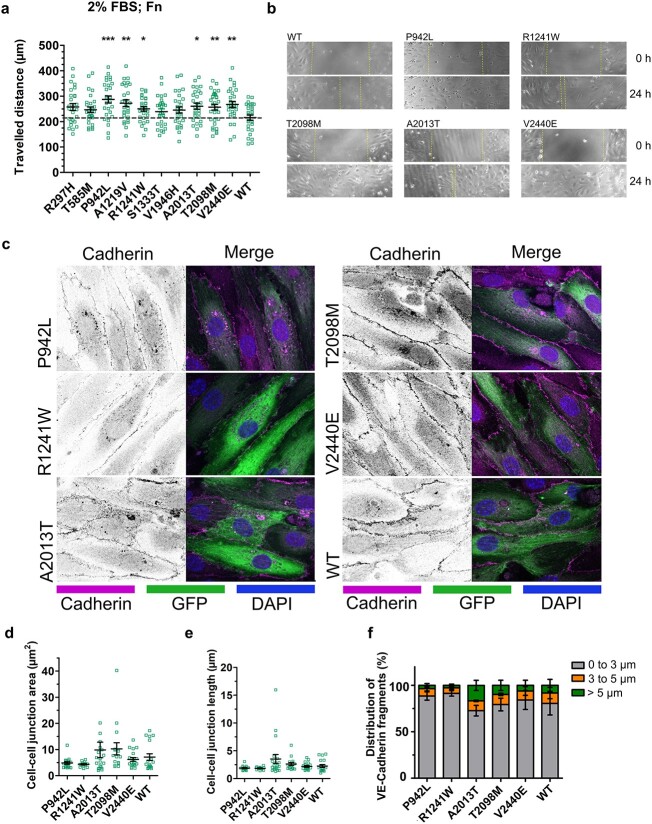
Impact of SCAD-associated talin-1 variants on epithelial cell monolayer integrity. HUVEC cells, subjected to talin-1 siRNA treatment, were transiently transfected to express GFP-talin-1 carrying SCAD-associated mutations. (a) The distance travelled (μm) by the cells to close the artificial wound, was calculated by analysing the scratched area at 0 h and after 24 h. n ~ 25 scratches from three independent experiments. The statistical analysis was done using t-test Mann–Whitney test; ^*^P < 0.05, ^*^^*^P < 0.01, ^*^^*^^*^P < 0.001. Data represent the mean values with SEM. (b) Representative images of the wound closure assay for the cells transfected with full-length talin WT, P942L, R1241W, T2098M, A2013T and V2440E in 0 h (upper) and after 24 h (lower) (c) representative images of the monolayer-cultured HUVEC depicting cadherin staining in the talin-transfected cell monolayer. (d–f) The distribution of VE-cadherin fragments in cell–cell junction in the monolayer of the transfected HUVEC cells with different talin constructs. The statistical analysis was done using group analysis two-way ANOVA Bonferroni test; ^*^P < 0.05, ^*^^*^P < 0.01, ^*^^*^^*^P < 0.001. Data represent the mean values with SEM.

As SCAD impacts the endothelial cells of the coronary artery [42–45], we next investigated the impact of talin-1 variants on endothelial cell behaviour. Human umbilical vein endothelial cells (HUVECs) were transfected with talin-1 siRNA, and efficient talin-1 knockdown was confirmed using Western Blot (WB) (Fig. S3). The talin-1 mutants were introduced into siRNA treated HUVECs via transient transfection. The talin re-expression levels were comparable to non-siRNA-treated cells (Fig. S3). No indications of proteolytic degradation due to possible folding defects were seen in the expressed talin-1 mutants as assessed via WB (Fig. S4). Neither vinculin or β1-integrin expression levels were drastically changed in response to the mutation as assessed via WB (Fig. S4). Following transfection, a wound healing assay in minimal serum conditions was conducted to measure the HUVEC cell migration, revealing that mutations P492L, A1219V, R1241W, A2013T, T2098M, and V2440E significantly increased cell migration speed. In contrast, mutations R297H, T585M, S1333T and V1946H had no noticeable influence (Fig. 3a and b).

Finally, we sought to test whether the talin variants compromise the integrity of the endothelial layer. To do this we investigated the impact of talin variants on cell–cell junctions in endothelial cells. Cell–cell junction integrity was evaluated using VE-cadherin staining (Fig. 3c–f) and analysis was performed for mutants P492L, R1241W, A2013T, T2098M, and V2440E. These five mutants were selected because they significantly affected cell migration velocity in both MKF and HUVEC cells compared to wild-type talin. Quantification of cadherin staining revealed that the average cell–cell junction area (Fig. 3d) was slightly larger in cells carrying the A2013T (~10 μm^2^; *P* = 0.80) and T2098M (~10 μm^2^; *P* = 0.07) mutations compared to those expressing wild-type talin (~7 μm^2^) but statistical significance was not met. Similarly, cell–cell junction fragment lengths were extended in cells with the A2013T (~3.5 μm; *P* = 0.19) and T2098M (~2.5 μm; *P* = 0.15) mutations, although these changes were subtle and not statistically different from wild-type talin (~1.95 μm) (Fig. 3e). As cell morphology was analysed, we did not see significant changes in cell size, circularity or aspect ratio between the samples (Fig. S5). We attempted to measure the effects of monolayer permissiveness as a result of these mutations, however these experiments were unsuccessful. Correct talin expression level seems to be essential for robust endothelial monolayer formation [6] and hence this experimental setup using (transient) overexpression of talin prevented experiments measuring monolayer permissiveness.

Total VE-cadherin expression levels showed no statistical differences in HUVEC cells transfected with the talin mutants, as determined by WB (Fig. S6). Upon closer examination of VE-cadherin junctions within cell–cell junctions, we quantified VE-cadherin fragments in three size groups: 0–3 μm, 3–5 μm, and above 5 μm. It was observed that the A2013T mutant exhibited a slightly higher proportion of fragments in the size range of 5 μm and above when compared to the wild-type; however, these differences were not statistically significant (*P* > 0.05). None of the other mutants showed notable differences compared to the wild-type. These findings suggest that SCAD-associated talin variants, including A2013T, have very modest impact on the integrity of cell–cell junctions *in vitro*.

## Discussion

The vast amount of sequencing data now available and being generated has necessitated the development of pathogenic prediction tools to distinguish between variations in genes that are likely benign single nucleotide polymorphisms (SNP) and those that are potentially pathogenic. Here we report that these tools may miss pathogenic variants in proteins that are multi-functional and involved in many cellular processes.

For proteins that are not classified as disease-associated, new variants are not immediately followed up. This was evident in our recent study on the talin-1 P229S variant [25] where the identification of a *de novo* variant in talin-1 was only included as a footnote on the patient’s genetic report “However, the *TLN1* gene has not yet been associated with any well-established human clinical disease or OMIM phenotypes at this time… Periodic reassessment of the medical literature is recommended to monitor for additional evidence of gene-disease association of the *TLN1* gene” for over a year until it was investigated following our analysis of *TLN1* mutations in the COSMIC database, where we fortuitously analysed a P229L mutation [24]. Such gaps can delay finding the proper treatment/correct diagnosis.

From the careful work of Turley and co-workers [22] a genetic link between a talin-1 variant, A2013T, and spontaneous coronary artery dissection (SCAD) was firmly established. This link between talin-1 and SCAD motivated the team to perform a subsequent analysis of genetic data from other SCAD patients, identifying a whole set of talin-1 mutants that are found in SCAD patients [46]. Whilst, only the A2013T variant is confirmed as pathogenic, the other nine variants identified in the SCAD cohort may be linked to increased risk of SCAD. In this work we set out to explore the A2013T variant and other SCAD-associated variants. Here we report the striking finding that variants in talin-1, including the A2013T variant, can get classified as benign by pathogenic prediction servers such as PROVEAN and AlphaMissense yet still be pathogenic (Table 1). Furthermore, the effects of these mutations on talin-1 domains can be detected at the protein level and at the cellular level.

Our study investigated how A2013T and the other SCAD-associated talin-1 mutations influence talin function and we report that many of the SCAD-associated mutations also cause measurable changes in talin-1 function. These effects are similar to those resulting from the A2013T variant supporting the idea that the other talin-1 mutations found in SCAD patients may play a role in SCAD pathology. Certain SCAD-associated talin-1 variants (P942L, R1241W, A2013T, T2098M, and V2440E) were observed to cause more prominent defects in cell behaviour and correlated with impacts on protein structure and folding. Molecular dynamics simulations and NMR spectroscopy suggested only modest changes in the structure of the majority of affected subdomains of talin (Table 2; Fig. 2a–c; Fig. S1). We observed that variants R1241W, A2013T and T2098M slightly decreased the thermal stability of the purified talin fragments (Table 2), mostly in line with the predictions made with the help of computational analysis (T2098M being an exception, where opposite effect was predicted by MD simulations and CD analysis).

Previous studies have shown that talin-dependent integrin activation not only plays a crucial role in cell-ECM adhesions but can also stabilise endothelial junctions and the endothelial barrier [15]. It has been also revealed that exon skipping in the talin-1 gene can result in loss of junctional integrity [16]. Furthermore, talin-1 has recently been shown to contribute to tissue mechanical homeostasis by sensing and responding to the stiffness of the extracellular matrix (ECM), a process that is important for maintaining the structural integrity and function of tissues [47]. Across our cell assays (Table 3; Fig. 3), we clearly detect functional defects caused by the talin-1 mutations. Changes in cellular behaviours suggest potential implications at the organismal level.

The mortality rate of SCAD ranges from 0.7% to 1% reinforcing the generally positive prognosis associated with SCAD [48]. However, the predictive power of genetic sequencing in the early detection of SCAD is currently limited. A key finding of this study is that the talin-1 A2013T variant, linked with familial SCAD, is currently mis-classified as benign by several pathogenicity predictors, which indicates that the classification of variants in proteins such as talin-1 might need to be recalibrated. This misclassification also suggests a possibility that there may be many other diseases caused by subtle variants in talin-1 that are currently overlooked due to the benchmarking of pathogenicity being established uniformly across all proteins. Talin is a multi-functional protein which interacts with numerous proteins and undergoes dramatic structural transitions in its rod domains, exhibiting switch-like behaviour, converting between folded and unfolded states to engage different protein partners [49]. Due to this structural plasticity each talin domain adopts different conformations and states, and so consideration of the domains as globular, folded entities obfuscates the dynamic nature of this protein scaffold. This might be a reason why what appear to be subtle changes in amino acid sidechain, as observed in the A2013T, can still cause a human phenotype.

Overall, we conclude that SCAD-associated talin variants are correctly folded, functional proteins which cause only mild phenotypes in cells. This is in agreement with the previous studies where disease-associated talin variants have shown mild cellular phenotypes, in line with the central role of talin as common adapter for numerous different integrins [24, 25]. This is also not surprising from the perspective of the disease characteristics, as SCAD typically presents in middle age, and therefore in development and early life these mutated proteins are likely able to perform the required functions. The exact triggers or changes that result in the manifestation of the condition are not fully known. However, it is plausible that age-related tissue stiffening could increase the mechanical stress on talin molecules [50]. Additionally, changes in the pressure or tension acting on talin [51, 52], or alterations in the proteins that interact with talin [25, 53, 54], could potentially influence the function of talin and contribute to the manifestation of the condition. The size of talin (2541 amino acids) and its large number of binding partners and conformational states leads us to presuppose that there might be numerous disease-causing variations in the *TLN1* gene and that a whole class of “talinopathies” might be revealed by re-evaluation of talin-1 variant pathogenicity factoring in talin’s myriad roles in mechanical signalling.

## Materials and methods

### Molecular dynamics simulations

PDB structures were used as starting conformations for MD: 3IVF for F2 (residues 208–306) [55], 1SJ8 for R1 (residues 487–656) [56], 2LQG for R4 (residues 910–1044) [57], 2L10 for R6 (residues 1205–1354) [57], 2KVP for R10 (residues 1822–1972) [58], 3DYJ for R11 (residues 1975–2140) [36] and 2JSW for R13 (residues 2300–2478) [59]. Structure preparation and visualisation were performed using PyMOL.

Alchemical free energy calculations were prepared using PMX [60] and performed with Gromacs [61] at Puhti supercomputer, CSC, Finland. The Amber99SB^*^–ILDN force field [62] and TIP3P water model in 0.15 M NaCl solution were used. Each system was energy minimised for 10 000 steps and then equilibrated for 1 ns using harmonic position restraints on all heavy atom of the protein. The temperature and pressure of the system was maintained at 298 K and 1 bar using Berendsen algorithm [63] for the system equilibration, while V-rescale [64] and Parrinello-Rahman [63] algorithms were used for equilibrium MD and non-equilibrium morphing simulations. Integration time step of 2 fs was used in all the simulations. Each state of the system was run for 100 ns equilibrium MD. 100 non-equilibrium morphing simulations were prepared for each physical state of the system, using snapshots captured from the equilibrium trajectories, linearly spaced from 10.9 to 100 ns. Fast non–equilibrium simulations were morphing the system from one state to another in 100 ps for change conserving mutations, and in 200 ps for charge changing mutations. A soft-core potential [65] was used. The whole calculation, including system preparation, was repeated at least three times and average free energy value was obtained.

### Structural modelling using AlphaFold

Structure models of mutants were generated using ColabFold [66] to run the structure prediction tool AlphaFold.

### Cell line, transfection, and Talin expression constructs

The *TLN1*−/−, *TLN2*−/− mouse kidney fibroblast (MKF) cell line, as previously described [37], were used in this study. Cells were cultured in a humidified incubator at 37°C with 5% CO_2_. High-glucose Dulbecco's modified Eagle medium (DMEM) supplemented with varying concentrations of fetal bovine serum (FBS) ranging from 0% to 10% was used in all experiments involving MKF cells.

Primary human umbilical vein endothelial cells (HUVECs) were obtained from PromoCell (Catalog number #C-12208; Germany). These cells were maintained at 37°C in a humidified atmosphere containing 5% CO2, using EMB-2 medium (#CC-3156; Lonza) supplemented with all necessary supplements (#CC-4176; Lonza) with 2% FBS. Both cell lines underwent regular testing to ensure they were free from mycoplasma contamination.

The expression constructs for cell culture experiments featured a modified pEGFP-C1 vector backbone (Clontech) which introduces an N-terminal EGFP-tag and included the following: full-length wild-type talin-1 (1–2541) and point mutations designated as R297H, T585M, P942L, A1219V, R1241W, S1333T, V1964I, A2013T, T2098M, V2440E. These constructs were provided as sequence-verified synthetic genes by GenScript (Piscataway, NJ, USA).

MKF cells were transfected via electroporation, utilising 6 μg of plasmid DNA per 10^6^ cells with the Neon transfection system (Thermo Fisher Scientific) using the following parameters: 1400 V, 30 ms, one pulse. HUVEC cells were also transfected using the same system, employing 20 μg of plasmid DNA per 2 × 10^6^ cells, with the following parameters: 1350 V, 30 ms, one pulse.

### siRNA transfection for HUVEC cells

Human talin-1 siRNAs were purchased as a custom mRNA of 25 oligonucleotides (nucleotides 4395–4419: CCAAGAACGGAAACCUG CCAGAGUU) (Horizon Discovery Ltd). The negative control siRNA was purchased from Thermo Fisher Scientific; #4390843). HUVEC cells were trypsinised and resuspended in 2 × 10^6^ cells in resuspension buffer from the Neon kit. 1 nM siRNA and the talin plasmid were added followed by electroporation. After transfection, cells were immediately plated on dishes containing the complete media. 55 h later, cells were subjected to either migration or immunofluorescence assays as described.

### Cell migration rate analysis

The migration assay was performed on transfected MKF cells in three different medium and coverslip-coated conditions: 1) 10% FBS containing medium and using 10 μg/ml fibronectin coated coverslips; 2) medium without FBS (0%) and using 10 μg/ml fibronectin coated coverslips; 3) medium without FBS (0%) and using 10 μg/ml vitronectin coated coverslips. Fibronectin and vitronectin were affinity-purified from human serum product Octaplas as described earlier [67, 68].

Transfected MKF cells were incubated overnight in the cell culture incubator, trypsinised and plated on well-plates coated with fibronectin or vitronectin as mentioned above. Cells were allowed to attach for 90 min, after which the medium was changed. The time-lapse images captured with EVOS FL auto microscope (Thermo Fisher Scientific) were analysed manually (12 h) using ImageJ (Fiji) and MTrackJ plugin [69, 70] from three separate experiments. The persistence of cell movement was analysed using ImageJ (Fiji) by analysing ratio of the squared displacement (d^2^) to the length travelled by the cell, providing insight into how straight or persistent a cell’s movement is over time.

### Cell morphology

To assess cell morphology, the fixed and stained HUVEC monolayer was imaged using a fluorescence microscope. Images were captured at 60× magnification with a 5×5 tile configuration. Cell outlines were manually traced using the freehand tool in ImageJ (Fiji) software. For each cell, the area, circularity and aspect ratio were measured.

### Wound closure assay

Transfected MKF cells were incubated in high confluency (90%) in a 24-well plate for 48 h in the cell culture incubator. The artificial wound (approximately 800 μm) was created using a 100 μl pipette tip. After, the wells were washed with media to remove dead and detached cells. The collective migration speed was observed using EVOS FL auto microscope. The images were analysed manually using ImageJ (Fiji) by using a freehand line tool measuring the distance that cells travelled in 12 h from the starting point. The assay was performed in two different medium conditions with 10% FBS containing medium and in 0% FBS medium.

### Immunofluorescence

HUVEC cells were transfected and plated on fibronectin-coated coverslips (30 μg/ml) for 55 h in the cell culture incubator for siRNA to affect after which cells were fixed with 4% paraformaldehyde, permeabilised and immunostained using standard protocol. The siRNA effect and recombinant talin expression were checked using western blots with talin-1 (97H6) (Mouse mAb, Lot CRT/17/98, Novus biologicals) primary antibodies. To investigate cell–cell junction integrity, VE-cadherin (Cell Signaling Technology, #2158, 1:100) was used as primary antibody and Alexa Fluor 568 goat anti-rabbit IgG (Life Technology A11011, 1:200) was used as secondary antibody (Table S1). Immunostained samples were imaged with Zeiss LSM800 laser scanning confocal microscope, mounted on inverted Zeiss Axio Observer.Z1 (Zeiss, Oberkochen, Germany) using Plan-Apochromat 63×/1.40, WD 0.19 mm oil immersion objective. The junctional integrity was quantified with IMARIS (Oxford Instruments 10.0.0) software. Signals were translated into surface objects, which further classified into two categories for the background and VE-cadherin using the machine learning for the filter type. The sizes of the VE-Cadherin signals were obtained by vantage plot function. 

### Protein expression and purification

For biochemical characterisation, previously reported pET151 plasmids for mouse talin-1 R4 (residues 913–1044), R11 (residues 1974–2140), and R13 (residues 2300–2482) (R4, [57], R11 [36], R13 [59] and codon optimised synthetic genes for R6 (residues 1206–1357) were used. The plasmids encoding talin mutants, R4 P942L, R6 R1241W, R11 A2013T, R11 T2098M and R13 V2440E were produced and sequence-verified by GeneArt. BL21 Star (DE3) *E. coli* were used.

To produce ^15^N-labelled proteins, BL21 Star (DE3) *E. coli* cells transformed with talin constructs were grown in 10 ml of LB + 100 μg/ml ampicillin at 37°C overnight which was used to inoculate 1 L 2 M9 minimal medium containing ^15^N-ammonium chloride and 100 μg/ml ampicillin. The 1 L cultures were incubated at 37°C until OD600 0.7–0.8, and then incubated overnight at 20°C following induction with 1 mM IPTG. The harvested cell pellets were resuspended in 20 mM Tris–HCl, pH 8, 500 mM NaCl, and 20 mM imidazole, and lysed by sonication. The proteins were purified from the cell lysate by nickel affinity chromatography using a 5 ml HisTrap HP column (GE Healthcare). Overnight dialysis was set up in either, 20 mM Tris–HCl, pH 8, 50 mM NaCl for R11 and R13, or 20 mM NaH_2_PO_4_, pH 6.5, 50 mM NaCl for R4 and R6 with AcTEV protease (Invitrogen) added to each to remove the His-tag. Following dialysis, the proteins were purified with a HiTrap Q HP anion exchange column (GE Healthcare) (R11 and R13 wild-types and mutants) or HiTrap SP HP cation exchange column (GE Healthcare) (R4 and R6 wild-types and mutants). Further details on the protein purification are available in [71].

### Nuclear magnetic resonance (NMR)

^15^N-labelled proteins were at 150 μM final concentration in 12 mM NaH_2_PO_4_, 6 mM Na_2_HPO_4_, pH 6.5, 50 mM NaCl, 2 mM DTT, 5%(v/v) D_2_O. NMR spectra were collected at 298 K using a Bruker Avance III 600 MHz NMR spectrometer equipped with CryoProbe. TopSpin and CCPN Analysis were used to process and analyse data [72].

### Circular dichroism (CD)

Protein samples were prepared at 400 μl of 0.5 mg/ml final concentration in the same buffer as for NMR in a quartz cuvette. Far-UV spectra were collected from 300 to 195 nm wavelength using a JASCO J-715 spectropolarimeter. CD signal changes at 222 nm were observed to see protein thermal unfolding between 20 and 90°C (R4 wild-type, R4 P942L, R6 wild-type and R6 R1241W) and between 20 and 95°C (R11 wild-type, R11 mutants, R13 wild-type and R13 V2440E).

## Supplementary Material

HMG-2024-CE-00301-Azizi_et_al_Supplementary_ddae120

## References

[1] Garcia MA , Nelson WJ, Chavez N. Cell–cell junctions organize structural and Signaling networks. Cold Spring Harb Perspect Biol 2018;10:a029181. 28600395 10.1101/cshperspect.a029181 PMC5773398

[2] Bachmann M , Kukkurainen S, Hytönen VP. et al. Cell adhesion by Integrins. Physiol Rev 2019;99:1655–1699. 31313981 10.1152/physrev.00036.2018

[3] Citi S , Fromm M, Furuse M. et al. A short guide to the tight junction. J Cell Sci 2024;137:jcs261776. 38712627 10.1242/jcs.261776 PMC11128289

[4] Mui KL , Chen CS, Assoian RK. The mechanical regulation of integrin-cadherin crosstalk organizes cells, signaling and forces. J Cell Sci 2016;129:1093–1100. 26919980 10.1242/jcs.183699 PMC4813297

[5] Aman J , Margadant C. Integrin-dependent cell-matrix adhesion in endothelial health and disease. Circ Res 2023;132:355–378. 36730379 10.1161/CIRCRESAHA.122.322332 PMC9891302

[6] Sidibé A , Mykuliak VV, Zhang P. et al. Acetyl-NPKY of integrin-β1 binds KINDLIN2 to control endothelial cell proliferation and junctional integrity. iScience 2024;27:110129. 38904068 10.1016/j.isci.2024.110129 PMC11187247

[7] Dissanayake WC , Shepherd PR. β-Cells retain a pool of insulin-containing secretory vesicles regulated by adherens junctions and the cadherin-binding protein p120 catenin. J Biol Chem 2022;298:102240. 35809641 10.1016/j.jbc.2022.102240 PMC9358467

[8] Dissanayake WC , Sorrenson B, Shepherd PR. The role of adherens junction proteins in the regulation of insulin secretion. Biosci Rep 2018;38:BSR20170989. 29459424 10.1042/BSR20170989 PMC5861323

[9] Parnaud G , Lavallard V, Bedat B. et al. Cadherin engagement improves insulin secretion of single human β-cells. Diabetes 2014;64:887–896. 25277393 10.2337/db14-0257

[10] Tixi W , Maldonado M, Chang Y-T. et al. Coordination between ECM and cell-cell adhesion regulates the development of islet aggregation, architecture, and functional maturation. elife 2023;12:e90006. 37610090 10.7554/eLife.90006 PMC10482429

[11] Canel M , Serrels A, Frame MC. et al. E-cadherin–integrin crosstalk in cancer invasion and metastasis. J Cell Sci 2013;126:393–401. 23525005 10.1242/jcs.100115

[12] Kilinc AN , Han S, Barrett LA. et al. Integrin-linked kinase tunes cell-cell and cell-matrix adhesions to regulate the switch between apoptosis and EMT downstream of TGFβ1. Mol Biol Cell 2021;32:402–412. 33405954 10.1091/mbc.E20-02-0092 PMC8098849

[13] Nasir Uddin SM , Sultana A, Fatima A. et al. Regulation of tight junction by cadherin adhesion and its implication in inflammation and cancer. In: Bhat AA, Haris M, Macha MA, Dhawan P (editors), Tight Junctions in Inflammation and Cancer. Singapore: Springer Nature, 2023, pp. 49–66.

[14] Weber GF , Bjerke MA, DeSimone DW. Integrins and cadherins join forces to form adhesive networks. J Cell Sci 2011;124:1183–1193. 21444749 10.1242/jcs.064618 PMC3115772

[15] Pulous FE , Grimsley-Myers CM, Kansal S. et al. Talin-dependent integrin activation regulates VE-cadherin localization and endothelial cell barrier function. Circ Res 2019;124:891–903. 30707047 10.1161/CIRCRESAHA.118.314560 PMC6521868

[16] Elefant N , Nikolopoulou PA, Papadaki VV. et al. Talin1 dysfunction is genetically linked to systemic capillary leak syndrome. MedRxiv [Preprint] 25 Oct 2022. 10.17.22280833.

[17] Yang C , Alfadhel M, Saw J. Spontaneous coronary artery dissection: latest developments and new Frontiers. Curr Atheroscler Rep 2020;22:49. 32734349 10.1007/s11883-020-00866-4

[18] Yang C , Offen S, Saw J. What is new in spontaneous coronary artery dissection? CJC Open 2024;6:417–424. 38487071 10.1016/j.cjco.2023.10.007 PMC10935686

[19] Brinza EK , Gornik HL. Fibromuscular dysplasia: advances in understanding and management. Cleve Clin J Med 2016;83:S45–S51. 27861117 10.3949/ccjm.83.s2.06

[20] Georges A , Bouatia-Naji N. The complex genetic basis of fibromuscular dysplasia, a systemic arteriopathy associated with multiple forms of cardiovascular disease. Clin Sci 2022;136:1241–1255. 10.1042/CS20210990 PMC9434409 36043395

[21] Schamroth Pravda N , Houri O, Kornowski R. et al. Clinical considerations during spontaneous coronary artery dissection in the post-partum period: a case report. Eur Heart J Case Rep 2023;7:ytad406. 37637097 10.1093/ehjcr/ytad406 PMC10448854

[22] Turley TN , Theis JL, Sundsbak RS. et al. Rare missense variants in TLN1 are associated with familial and sporadic spontaneous coronary artery dissection. Circ Genom Precis Med 2019;12:e002437. 30888838 10.1161/CIRCGEN.118.002437 PMC6625931

[23] Monkley SJ , Kostourou V, Spence L. et al. Endothelial cell talin1 is essential for embryonic angiogenesis. Dev Biol 2011;349:494–502. 21081121 10.1016/j.ydbio.2010.11.010 PMC3025397

[24] Azizi L , Cowell AR, Mykuliak VV. et al. Cancer associated Talin point mutations disorganise cell adhesion and migration. Sci Rep 2021;11:347. 33431906 10.1038/s41598-020-77911-4 PMC7801617

[25] Azizi L , Varela L, Turkki P. et al. Talin variant P229S compromises integrin activation and associates with multifaceted clinical symptoms. Hum Mol Genet 2022;31:4159–4172. 35861643 10.1093/hmg/ddac163 PMC9759328

[26] Adzhubei IA , Schmidt S, Peshkin L. et al. A method and server for predicting damaging missense mutations. Nat Methods 2010;7:248–249. 20354512 10.1038/nmeth0410-248 PMC2855889

[27] Adzhubei I , Jordan DM, Sunyaev SR. Predicting functional effect of human missense mutations using PolyPhen-2. Curr Protoc Hum Genet 2013;76:20. 10.1002/0471142905.hg0720s76 PMC4480630 23315928

[28] Kumar P , Henikoff S, Ng PC. Predicting the effects of coding non-synonymous variants on protein function using the SIFT algorithm. Nat Protoc 2009;4:1073–1081. 19561590 10.1038/nprot.2009.86

[29] Ng PC , Henikoff S. Predicting deleterious amino acid substitutions. Genome Res 2001;11:863–874. 11337480 10.1101/gr.176601 PMC311071

[30] Choi Y , Chan AP. PROVEAN web server: a tool to predict the functional effect of amino acid substitutions and indels. Bioinformatics 2015;31:2745–2747. 25851949 10.1093/bioinformatics/btv195 PMC4528627

[31] Ioannidis NM , Rothstein JH, Pejaver V. et al. REVEL: an ensemble method for predicting the pathogenicity of rare missense variants. Am J Hum Genet 2016;99:877–885. 27666373 10.1016/j.ajhg.2016.08.016 PMC5065685

[32] Takeda J-I , Nanatsue K, Yamagishi R. et al. InMeRF: prediction of pathogenicity of missense variants by individual modeling for each amino acid substitution. NAR Genom Bioinform 2020;2:lqaa038. 33543123 10.1093/nargab/lqaa038 PMC7671370

[33] Cheng J , Novati G, Pan J. et al. Accurate proteome-wide missense variant effect prediction with AlphaMissense. Science 2023;381:eadg7492. 37733863 10.1126/science.adg7492

[34] Ljungdahl A , Kohani S, Page NF. et al. AlphaMissense is better correlated with functional assays of missense impact than earlier prediction algorithms. bioRxiv [Preprint]. 2023 Oct 27:2023.10.24.562294.

[35] Gapsys V , Michielssens S, Seeliger D. et al. Accurate and rigorous prediction of the changes in protein free energies in a large-scale mutation scan. Angew Chem Int Ed 2016;55:7364–7368. 10.1002/anie.201510054 PMC5074281 27122231

[36] Gingras AR , Ziegler WH, Bobkov AA. et al. Structural determinants of integrin binding to the Talin rod. J Biol Chem 2009;284:8866–8876. 19176533 10.1074/jbc.M805937200 PMC2659244

[37] Theodosiou M , Widmaier M, Böttcher RT. et al. Kindlin-2 cooperates with Talin to activate integrins and induces cell spreading by directly binding paxillin. elife 2016;5:e10130. 26821125 10.7554/eLife.10130 PMC4749545

[38] Foolen J , Shiu J-Y, Mitsi M. et al. Full-length fibronectin drives fibroblast accumulation at the surface of collagen microtissues during cell-induced tissue morphogenesis. PLoS One 2016;11:e0160369. 27564551 10.1371/journal.pone.0160369 PMC5001707

[39] Liu S , Yang W, Li Y. et al. Fetal bovine serum, an important factor affecting the reproducibility of cell experiments. Sci Rep 2023;13:1942. 36732616 10.1038/s41598-023-29060-7 PMC9894865

[40] Pankov R , Cukierman E, Katz BZ. et al. Integrin dynamics and matrix assembly: tensin-dependent translocation of alpha(5)beta(1) integrins promotes early fibronectin fibrillogenesis. J Cell Biol 2000;148:1075–1090. 10704455 10.1083/jcb.148.5.1075 PMC2174533

[41] Sarkar A , LeVine DN, Kuzmina N. et al. Cell migration driven by self-generated integrin ligand gradient on ligand-labile surfaces. Curr Biol 2020;30:4022–4032.e5. 32916117 10.1016/j.cub.2020.08.020 PMC7578120

[42] Cano-Castellote M , Afanador-Restrepo DF, González-Santamaría J. et al. Pathophysiology, diagnosis and treatment of spontaneous coronary artery dissection in Peripartum women. J Clin Med 2022;11:6657. 36431134 10.3390/jcm11226657 PMC9692787

[43] Katz AE , Ganesh SK. Advancements in the genetics of spontaneous coronary artery dissection. Curr Cardiol Rep 2023;25:1735–1743. 37979122 10.1007/s11886-023-01989-1 PMC10810930

[44] Lee WE , Genetzakis E, Figtree GA. Novel strategies in the early detection and treatment of endothelial cell-specific mitochondrial dysfunction in coronary artery disease. Antioxidants (Basel) 2023;12:1359. 37507899 10.3390/antiox12071359 PMC10376062

[45] Tweet MS , Miller VM, Hayes SN. The evidence on Estrogen, progesterone, and spontaneous coronary artery dissection. JAMA Cardiol 2019;4:403–404. 30969320 10.1001/jamacardio.2019.0774

[46] Turley TN , O’Byrne MM, Kosel ML. et al. Identification of susceptibility loci for spontaneous coronary artery dissection. JAMA Cardiol 2020;5:929–938. 32374345 10.1001/jamacardio.2020.0872 PMC7203673

[47] Chanduri MVL , Kumar A, Weiss D. et al. Mechanosensing through Talin 1 contributes to tissue mechanical homeostasis. bioRxiv [Preprint]. 2024 Jan 26:2023.09.03.556084.

[48] Adams C , He M, Hughes I. et al. Mortality in spontaneous coronary artery dissection: a systematic review and meta-analysis. Catheter Cardiovasc Interv 2021;98:1211–1220. 33491851 10.1002/ccd.29488

[49] Guo Y , Yan J, Goult BT. Mechanotransduction through protein stretching. Curr Opin Cell Biol 2024;87:102327. 38301379 10.1016/j.ceb.2024.102327

[50] Kohn JC , Lampi MC, Reinhart-King CA. Age-related vascular stiffening: causes and consequences. Front Genet 2015;06:112. 10.3389/fgene.2015.00112 PMC4396535 25926844

[51] Goult BT , Brown NH, Schwartz MA. Talin in mechanotransduction and mechanomemory at a glance. J Cell Sci 2021;134:jcs258749. 34708856 10.1242/jcs.258749 PMC8697387

[52] Wang Y , Zhang X, Tian J. et al. Talin promotes integrin activation accompanied by generation of tension in Talin and an increase in osmotic pressure in neurite outgrowth. FASEB J 2019;33:6311–6326. 30768370 10.1096/fj.201801949RR

[53] Chen C , Manso AM, Ross RS. Talin and Kindlin as integrin-activating proteins: focus on the heart. Pediatr Cardiol 2019;40:1401–1409. 31367953 10.1007/s00246-019-02167-3 PMC7590617

[54] Zhao Y , Lykov N, Tzeng C. Talin-1 interaction network in cellular mechanotransduction (review). Int J Mol Med 2022;49:60. 35266014 10.3892/ijmm.2022.5116 PMC8930095

[55] Elliott PR , Goult BT, Kopp PM. et al. The structure of the Talin head reveals a novel extended conformation of the FERM domain. Structure 2010;18:1289–1299. 20947018 10.1016/j.str.2010.07.011 PMC2977851

[56] Papagrigoriou E , Gingras AR, Barsukov IL. et al. Activation of a vinculin-binding site in the Talin rod involves rearrangement of a five-helix bundle. EMBO J 2004;23:2942–2951. 15272303 10.1038/sj.emboj.7600285 PMC514914

[57] Goult BT , Zacharchenko T, Bate N. et al. RIAM and vinculin binding to Talin are mutually exclusive and regulate adhesion assembly and turnover. J Biol Chem 2013;288:8238–8249. 23389036 10.1074/jbc.M112.438119 PMC3605642

[58] Goult BT , Gingras AR, Bate N. et al. The domain structure of Talin: residues 1815–1973 form a five-helix bundle containing a cryptic vinculin-binding site. FEBS Lett 2010;584:2237–2241. 20399778 10.1016/j.febslet.2010.04.028 PMC2887493

[59] Gingras AR , Bate N, Goult BT. et al. The structure of the C-terminal actin-binding domain of Talin. EMBO J 2008;27:458–469. 18157087 10.1038/sj.emboj.7601965 PMC2168396

[60] Gapsys V , Michielssens S, Seeliger D. et al. Pmx: automated protein structure and topology generation for alchemical perturbations. J Comput Chem 2015;36:348–354. 25487359 10.1002/jcc.23804 PMC4365728

[61] Abraham MJ , Murtola T, Schulz R. et al. GROMACS: high performance molecular simulations through multi-level parallelism from laptops to supercomputers. SoftwareX 2015;1-2:19–25.

[62] Lindorff-Larsen K , Piana S, Palmo K. et al. Improved side-chain torsion potentials for the amber ff99SB protein force field. Proteins 2010;78:1950–1958. 20408171 10.1002/prot.22711 PMC2970904

[63] Berendsen HJC , Postma JPM, van Gunsteren WF. et al. Molecular dynamics with coupling to an external bath. J Chem Phys 1984;81:3684–3690.

[64] Bussi G , Donadio D, Parrinello M. Canonical sampling through velocity rescaling. J Chem Phys 2007;126:014101. 17212484 10.1063/1.2408420

[65] Gapsys V , Seeliger D, de Groot BL. New soft-Core potential function for molecular dynamics based alchemical free energy calculations. J Chem Theory Comput 2012;8:2373–2382. 26588970 10.1021/ct300220p

[66] Mirdita M , Schütze K, Moriwaki Y. et al. ColabFold: making protein folding accessible to all. Nat Methods 2022;19:679–682. 35637307 10.1038/s41592-022-01488-1 PMC9184281

[67] Poulouin L , Gallet O, Rouahi M. et al. Plasma fibronectin: three steps to purification and stability. Protein Expr Purif 1999;17:146–152. 10497080 10.1006/prep.1999.1103

[68] Yatohgo T , Izumi M, Kashiwagi H. et al. Novel purification of vitronectin from human plasma by heparin affinity chromatography. Cell Struct Funct 1988;13:281–292. 2460263 10.1247/csf.13.281

[69] Meijering E , Dzyubachyk O, Smal I. Methods for cell and particle tracking. Methods Enzymol 2012;504:183–200. 22264535 10.1016/B978-0-12-391857-4.00009-4

[70] Schneider CA , Rasband WS, Eliceiri KW. NIH image to ImageJ: 25 years of image analysis. Nat Methods 2012;9:671–675. 22930834 10.1038/nmeth.2089 PMC5554542

[71] Khan RB , Varela L, Cowell AR. et al. Biochemical characterization of the integrin Interactome. Methods Mol Biol 2021;2217:115–147. 33215380 10.1007/978-1-0716-0962-0_9

[72] Skinner SP , Goult BT, Fogh RH. et al. Structure calculation, refinement and validation using CcpNmr analysis. Acta Crystallogr D Biol Crystallogr 2015;71:154–161. 25615869 10.1107/S1399004714026662 PMC4304695

