# Supplementary material for: Talin-1 variants associated with spontaneous coronary artery dissection (SCAD) highlight how even subtle changes in multi-functional scaffold proteins can manifest in disease
Source: Hum Mol Genet. 2024 Aug 21;33(21):1846–57. doi: 10.1093/hmg/ddae120 (PMC11540920; doi:10.1093/hmg/ddae120)
Supplement: HMG-2024-CE-00301-Azizi_et_al_Supplementary_ddae120 [file hmg-2024-ce-00301-azizi_et_al_supplementary_ddae120.docx]

## Supplementary information for

## Talin-1 variants associated with spontaneous coronary artery dissection (SCAD) highlight how even subtle changes in multi-functional scaffold proteins can manifest in disease

Latifeh Azizi^1#^, Yasumi Otani^2#^, Vasyl V. Mykuliak^1^, Benjamin T. Goult^2,*^, Vesa P. Hytönen^1,3,*^, Paula Turkki^1,3,*^

^1^Faculty of Medicine and Health Technology, Tampere University, Tampere, Finland.

^2^Department of Biochemistry, Cell & Systems Biology, Institute of Systems, Molecular & Integrative Biology, University of Liverpool, Crown Street, Liverpool L69 7ZB, U.K.

^3^Fimlab Laboratories, Tampere, Finland.

^#^These authors contributed equally and share the 1^st^ authorship

*Shared last authorship

To whom correspondence may be addressed. Email: [b.t.goult@liverpool.ac.uk](mailto:b.t.goult@liverpool.ac.uk), [vesa.hytonen@tuni.fi](mailto:vesa.hytonen@tuni.fi) or [paula.turkki@tuni.fi](mailto:paula.turkki@tuni.fi)


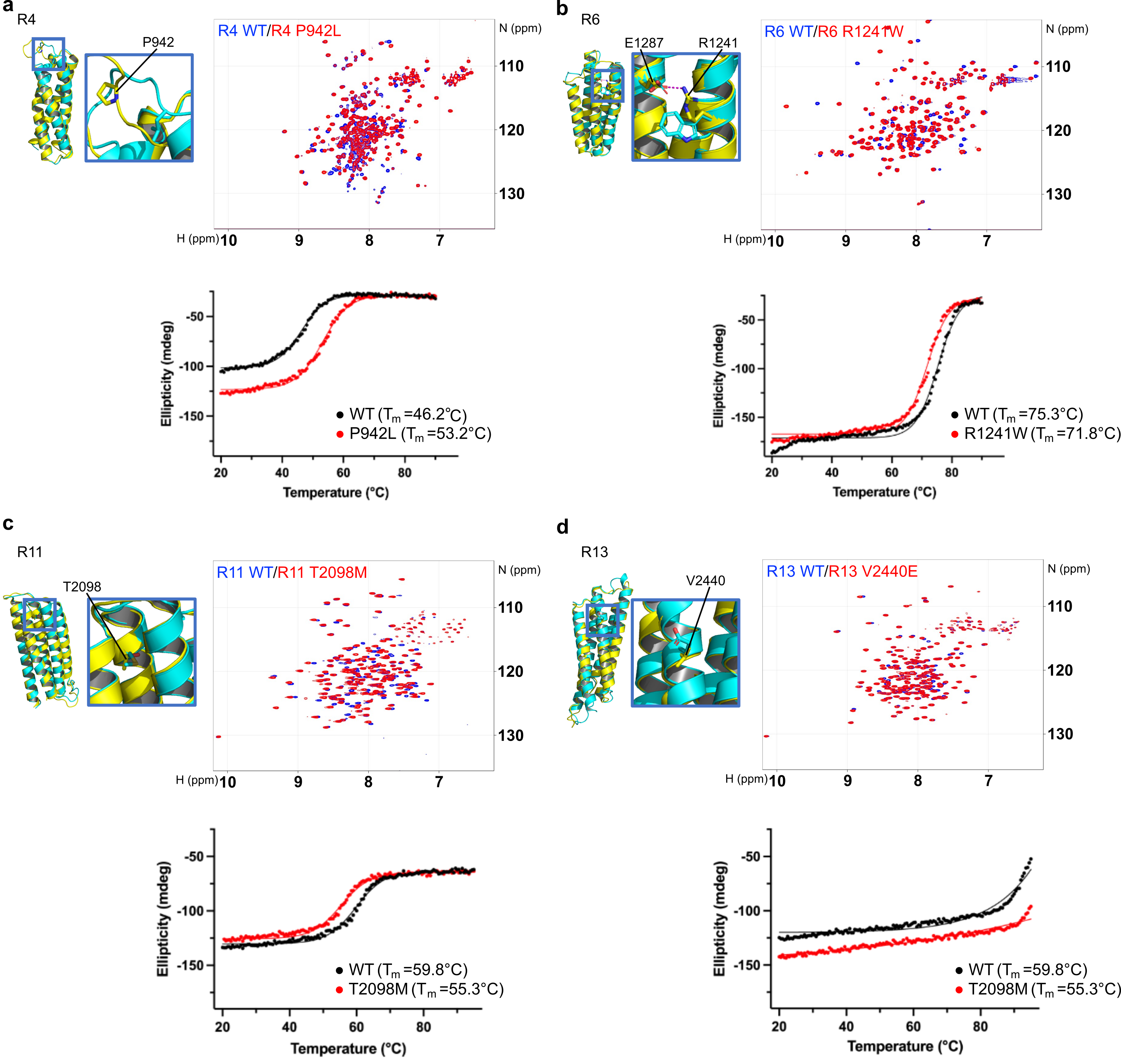


**Figure S1.** **Influence of SCAD-associated talin-1 variants on folding of talin domains.** **(a-d)** Biophysical analysis of each variant. (left) For each variant the structure of the wildtype (yellow) and mutant (cyan) talin rod domain are shown, (right) the corresponding ^1^H,^15^N-HSQC spectra of ^15^N-labelled talin-1 domain WT (blue) and mutant (red), and (bottom) Melting curves of talin-1 rod domains WT (black) and mutants (red). The melting temperature (T_m_) is shown in the legend. **(a)** R4 WT (yellow, PDB 2LQG (Goult et al., 2013)) and P942L (cyan), **(b)** R6 WT (yellow, PDB 2L10 (Goult et al., 2013)) and R1241W (cyan), the distance of charged residues of E1287 and R1241 in WT was 2.5 Å (magenta). **(c)** R11 WT (yellow, PDB 3DYJ (Gingras et al., 2009)) and T2098M (cyan) and **(d)** R13 WT (yellow, PDB 2JSW (Gingras et al., 2008)) and V2440 (cyan).


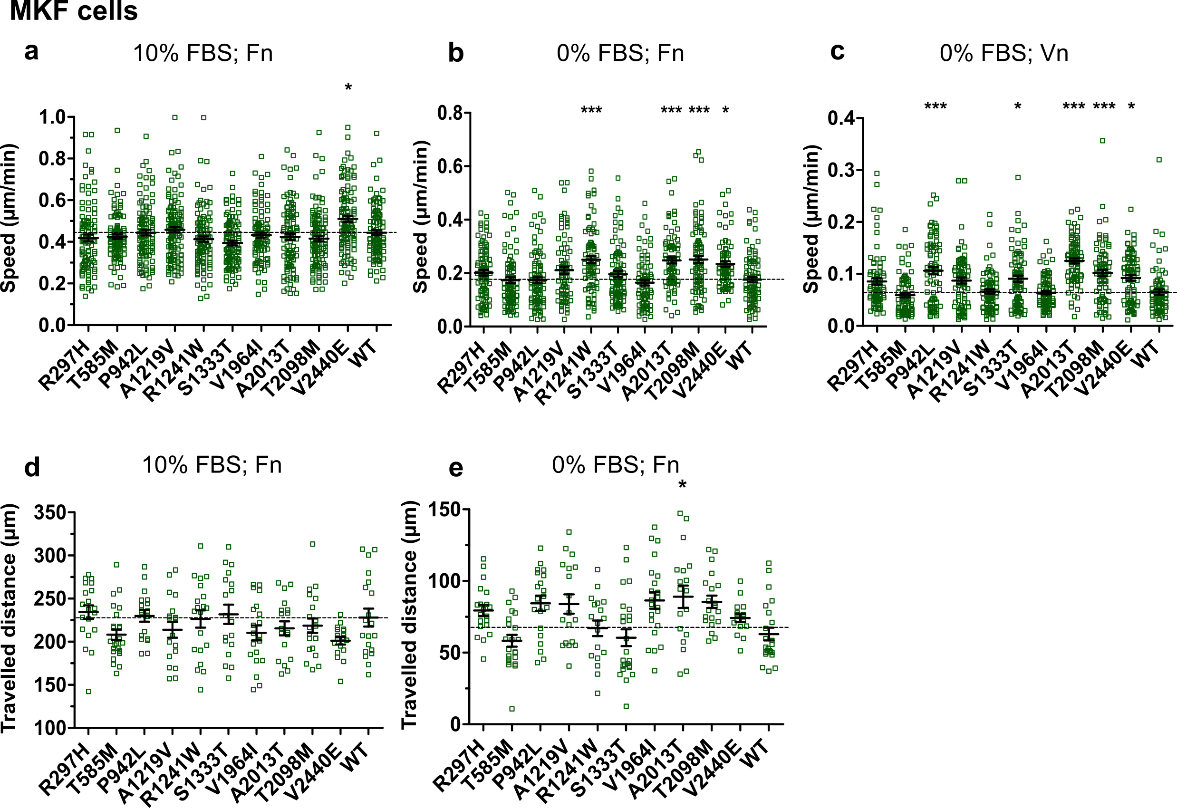


**Figure S2. Influence of SCAD-associated talin-1 variants on cell movement.** **(a,b,c)** *TLN1 -/-TLN2 -/-* MKF cells were transiently transfected to assess how each talin mutation influences the random migration velocity. n ~ 80 cells pooled from three independent experiments for each condition. The statistical significance was analysed by one-way ANOVA and Bonferroni test: *P<0.05, **P<0.01, ***P<0.001. **(d, e)** Distance travelled (μm) by cells to close an artificial wound were calculated by analysing the travelled distance from 0 h to 12 h for full-length talin WT and variants. n ~ 25 scratches from three independent experiments. The statistical analysis was done using t-test Mann–Whitney test; *P < 0.05, **P < 0.01, ***P < 0.001. Data represent the mean values with SEM.


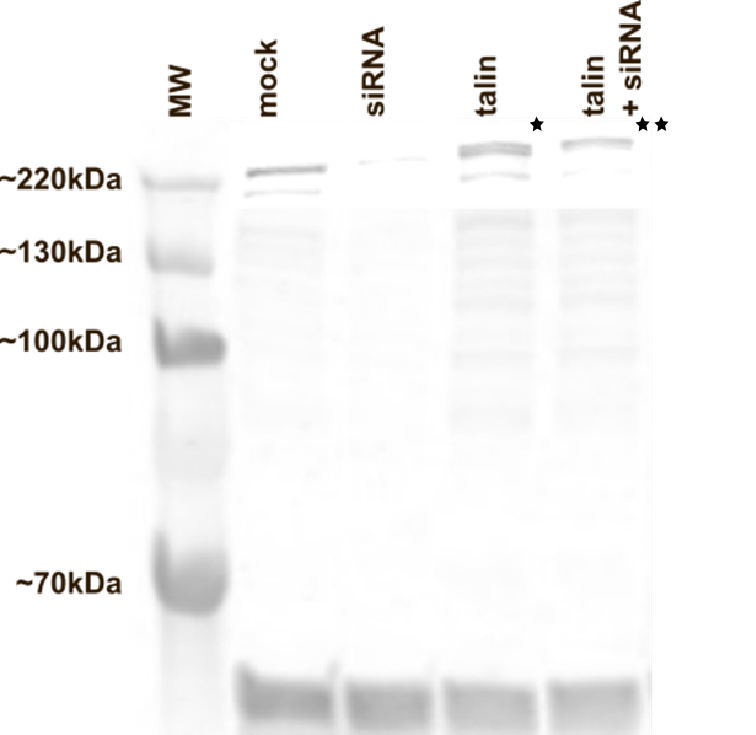


**Figure S3.** **Silencing of talin-1 using siRNA.** Western Blot analysis to assess efficacy of talin-siRNA transfection after 55 h. Anti-talin (97H6) was used to detect talin. Mock=HUVEC cells; siRNA=cells transfected only with siRNA; talin=cells transfected with 20 µg of talin full-length WT DNA; talin+siRNA= co-transfection of 20 µg of talin full-length WT plasmid with the talin-siRNA. The recombinant talin band is indicated by two stars (**) and can be distinguished from the endogenous and recombinant talin bands (indicated by one star (*)) due to the mobility shift caused by EGFP fusion partner present in the recombinant talin form.


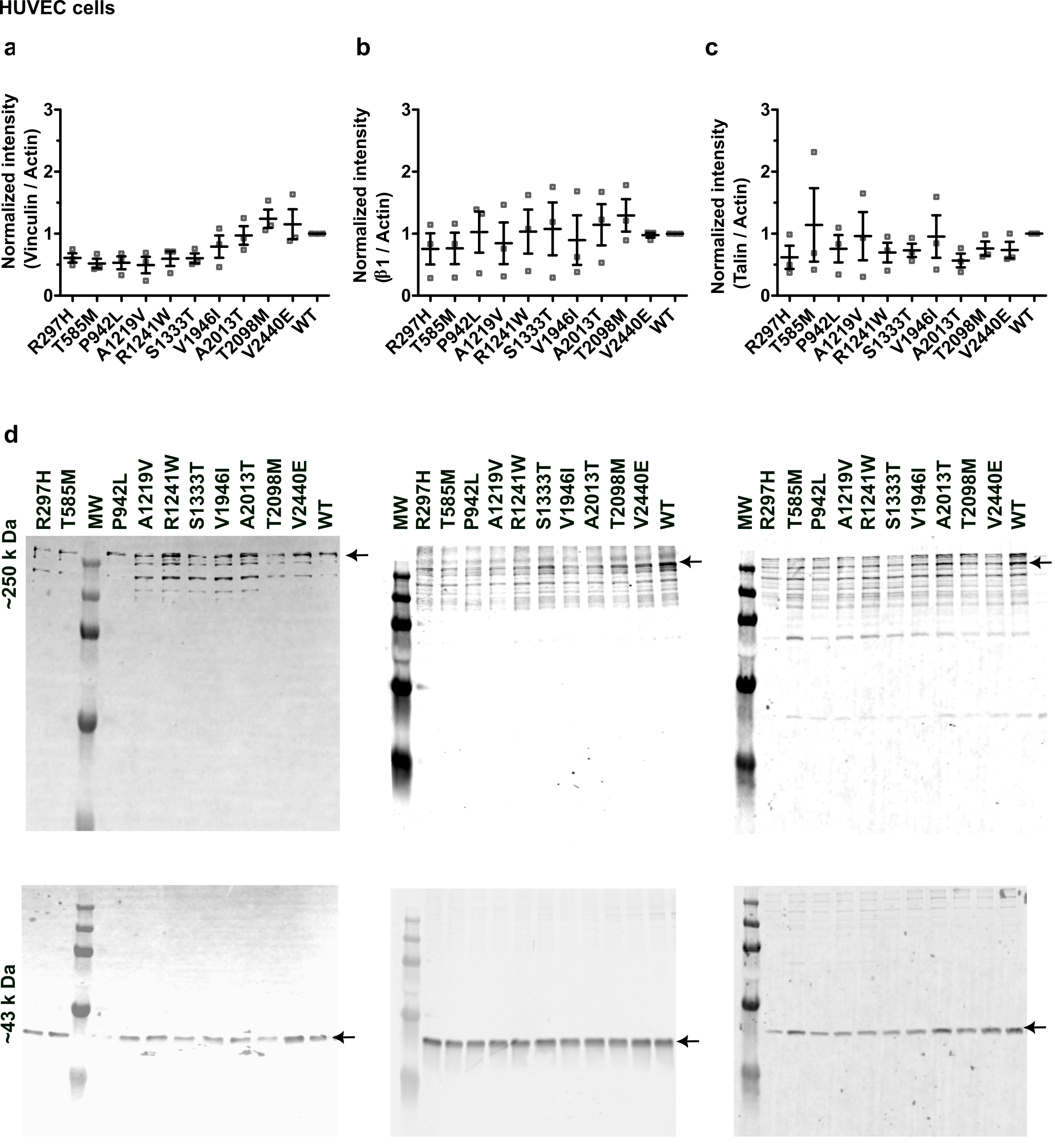


**Figure S4.** **Determination of protein expression levels in HUVEC cells transfected with SCAD-associated talin-1 variants.** Band intensity from western blot analysis for **(a)** vinculin **(b)** β1-integrin **(c,d).** Talin-1 blotted with antibody 97H6.


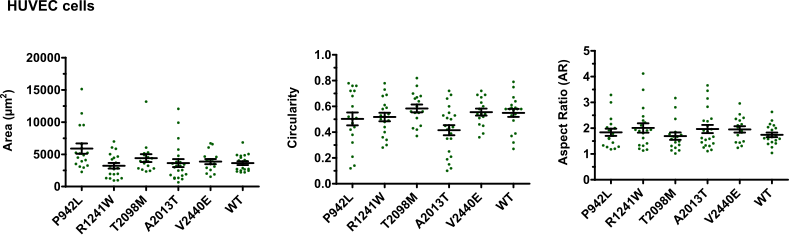


**Figure S5.** **SCAD-associated talin-1 variants do not compromise HUVEC cell spreading.** Cell area, circularity and aspect ratio were measured in cells expressing full-length versions of talin WT and its variants. ~25 cells pooled from three independent experiments were analysed. Statistical analysis was performed using the Mann–Whitney t-test; *P < 0.05, **P < 0.01, ***P < 0.001. Data are presented as mean values with standard error of the mean (SEM).


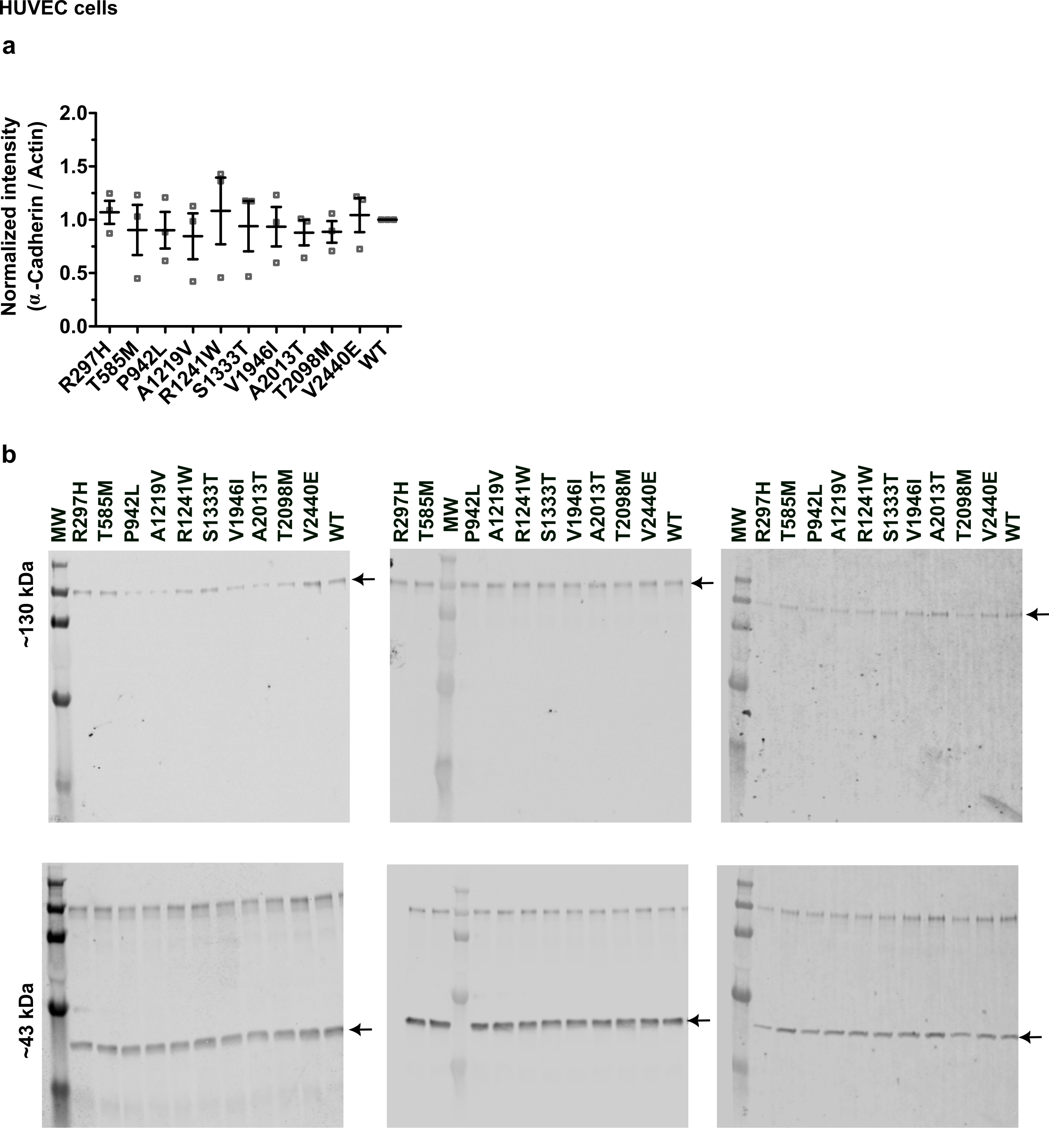


**Figure S6.** **Determination of VE-cadherin expression levels in HUVEC cells expressing talin mutants.** **(a)** Quantification of Western Blots showing cadherin expression level (Cadherin/actin). **(b)** Western Blots used for quantification of cadherin expression. Blots are immunolabelled against cadherin (top) and actin (bottom).

**Table S1.** Antibodies used in this study. Application: WB = Western blot; IF = immunofluorescence.

| **Antibody** | **Manufacturer** | **Dilution** |
| --- | --- | --- |
| GFP | Sicgen AB0020–200 | 1:1000 WB |
| Vinculin | Merck, clone hVIN, V9131, RRID:AB_477629 | 1:1000 WB |
| Integrin β1 (D6S1W) | Cell Signaling Technology, #34971 | 1:1000 WB |
| talin-1 (97H6) | Mouse mAb, Lot CRT/17/98, Novus biologicals | 1:1000 WB |
| VE-Cadherin Rabbit Ab | Cell Signaling Technology, Lot: 3 | 1:100 IF  1:1000 WB |
| Actin | Millipore, MAB 1501R, RRID: AB_2223041 | 1:2000 WB |
| IRDye® 800CW | LI COR, Goat a-Mouse, Lot# C80911-15 | 1:20 000 WB |
| IRDye® 800CW | LI COR, Donkey a-Goat, Lot# C90619-05 | 1:20 000 WB |
| IRDye® 680RD | LI COR, Goat a-Rabbit, Lot# C80911-15 | 1:20 000 WB |
| Alexa Fluor 568 goat anti–rabbit IgG | Life Technologies A11011 | 1:200 IF |

**Supplementary References**

**Gingras, A. R., Bate, N., Goult, B. T., Hazelwood, L., Canestrelli, I., Grossmann, J. G., Liu, H., Putz, N. S. M., Roberts, G. C. K., Volkmann, N., et al.** (2008). The structure of the C‐terminal actin‐binding domain of talin. *EMBO J.* **27**, 458–469.

**Gingras, A. R., Ziegler, W. H., Bobkov, A. A., Joyce, M. G., Fasci, D., Himmel, M., Rothemund, S., Ritter, A., Grossmann, J. G., Patel, B., et al.** (2009). Structural determinants of integrin binding to the talin rod. *J. Biol. Chem.* **284**, 8866–8876.

**Goult, B. T., Zacharchenko, T., Bate, N., Tsang, R., Hey, F., Gingras, A. R., Elliott, P. R., Roberts, G. C. K., Ballestrem, C., Critchley, D. R., et al.** (2013). RIAM and vinculin binding to talin are mutually exclusive and regulate adhesion assembly and turnover. *J. Biol. Chem.* **288**, 8238–8249.
